# Individual Treatment Decisions for Central Neurocytoma

**DOI:** 10.3389/fneur.2020.00834

**Published:** 2020-08-12

**Authors:** Song Han, Zuocheng Yang, Yakun Yang, Xueling Qi, Changxiang Yan, Chunjiang Yu

**Affiliations:** ^1^Department of Neurosurgery, Sanbo Brain Hospital, Capital Medical University, Beijing, China; ^2^Department of Pathology, Sanbo Brain Hospital, Capital Medical University, Beijing, China

**Keywords:** central neurocytoma, complete resection, partial resection, overall survival, progression-free survival, adjuvant radiotherapy

## Abstract

**Objective:** Central neurocytomas (CNs) are rare, and this has resulted in a paucity of information and a lack of clarity regarding their optimal management. This study aimed to explore individual treatment strategies for CNs and the benefits of these strategies for patients.

**Methods:** This single-center study retrospectively analyzed data from 67 patients with CNs who underwent surgery. Based on the extent of resection, patients were divided into complete and incomplete resection groups. The patients were followed, and overall survival (OS) and progression-free survival (PFS) were determined.

**Results:** Of 55 patients (82.1%) who underwent complete resections, 24 received radiotherapy (24/55, 43.6%). Of 12 patients who underwent incomplete resections, 9 (9/12, 75.0%) received radiotherapy. The OS (*p* = 0.003) and PFS (*p* = 0.006) intervals were significantly longer in the complete resection group than in the incomplete resection group. Postoperative radiotherapy did not affect OS (*p* = 0.129) or PFS (*p* = 0.233) in the complete resection group. In the incomplete resection group, postoperative adjuvant radiotherapy prolonged patient survival significantly (*p* = 0.021). PFS was significantly longer among patients who underwent complete resection without radiotherapy than in those who underwent incomplete resection followed by radiotherapy (*p* = 0.034). Functional dependence on admission, which was defined as a Karnofsky Performance Status score <70, was an independent risk factor associated with long-term survival in patients with CN. Postoperative complications were not associated with the amount of tumor resected. The prognosis of patients aged ≥ 50 years was relatively poor. The atypical CN recurrence rate was relatively high (7.8%).

**Conclusions:** To protect function as much as possible, complete tumor resection should be the first choice of treatment for CN. After gross total resection, adjuvant radiotherapy is not acceptable. Postoperative adjuvant radiotherapy improves the prognosis of patients who have undergone incomplete tumor resections. Adjuvant radiotherapy is not recommended after complete resections of atypical CNs, and close follow-up with imaging is required. Our findings can help guide decision-making regarding the treatment of CNs and could potentially maximize the benefits of treatment for patients with CN.

## Introduction

In 1982, Hassoun et al. first described central neurocytoma (CN) as a rare ventricular tumor; subsequently, CN has become a definitive clinical and pathologic entity based on information describing its clinical, radiologic, and histopathologic characteristics (1). Of the patients affected by CN, 70.0% are aged between 20 and 40 years, and its incidence does not show any sex preponderance (2, 3). About 25.0% of the tumors are “atypical CNs” (2–4); these are more aggressive and are characterized by a Ki-67 index > 2.0–3.0%, and they may have infiltrative margins, higher levels of mitotic activity, nuclear atypia, necrosis, and vascular proliferation (5–7).

At present, surgery is the mainstay of CN treatment. Some researchers consider that the tumor's resection range is the most important prognostic factor (4), and that complete resection of CN could achieve better control of local tumor recurrence (5, 6); however, the correlation between total tumor resection and overall survival (OS) remains controversial (3, 7, 8). In addition, CNs are located deep within the brain close to key structures, including the fornix, thalamus, caudate nucleus, and striate veins, and it is difficult to resect these tumors completely (9), which increases morbidity and mortality rates (9–11). Some investigators considered that radiotherapy could control the recurrence of CNs that cannot be completely resected (7, 12). Imber et al. summarized 20 years of experience in treating CN, and they considered that adjuvant radiotherapy after subtotal tumor removal can improve patients' progression-free survival (PFS) (2). However, adjuvant radiotherapy is associated with patient complications. Cognitive dysfunction and memory impairment induced by radiotherapy have significant negative impacts on patients' quality of life (2). Moreover, adjuvant radiotherapy does not improve patients' Karnofsky Performance Status (KPS) scores after complete tumor resection (13), and whether it is necessary after complete resection of CN remains controversial (14–17). Hence, the administration of radiotherapy to treat CN requires a comprehensive evaluation.

In view of the lack of clarity about CN treatment, we examined the survival, complications, and the long-term functional status of patients with CN following treatment to determine whether adjuvant radiotherapy after complete resection is required, whether incomplete resection and adjuvant radiotherapy could replace complete resection given the difficulties and disabilities patients experience following complete tumor resection, and whether additional treatment is needed after the complete resection of atypical CNs.

## Methods

### Patients

From June 2008 to August 2019, patients who had neurocytomas were diagnosed and treated at Capital Medical University's Sanbo Brain Hospital were examined retrospectively. Data describing the patients' ages, sexes, clinical manifestations, and pathology and imaging findings were extracted, and the patients' follow-up data were obtained by reviewing the hospital's files, through telephone conversations, and by evaluating the data from the patients' follow-up assessments. Included in follow-up visits were imaging and functional evaluations: cerebral CT reexamination was conducted 1 week following discharge to confirm if there was hydrocephalus; cerebral MRI reexamination was needed 3 months following operation to confirm if there was tumor left after resection; the reexamination frequency was determined based on whether there was tumor residue. If there was residual tumor, radiotherapy was recommended; cerebral MRI reexamination was conducted every 3 months and lengthened to every 6 months once the condition was stabilized. If there was no residual tumor, cerebral MRI reexamination went every 6 months before 2 years following operation, then cerebral MRI reexamination went every 1 year. There were functional evaluations to every imaging reexamination.

### Pathologic Centralized Evaluation

The slides of the original surgical specimens were collected and reviewed blindly by a group of 3 neuropathologists. The histologic features were evaluated for each neurocytoma: presence or absence of nuclear atypia; presence or absence of vascular proliferation; the mitotic index based on counting 10 successive high-power fields (HPFs), presence or absence of necrosis and calcifications; the mitotic index based on counting 10 high-power fields (HPFs). Seven cases of extraventricular neurocytomas were excluded. A Ki-67 index > 2.0% for tumor with necrosis, higher levels of mitotic activity, vascular proliferation, or nuclear atypia was considered to indicate an atypical CN (4, 18).

### Imaging Evaluations

The tumors' diameters were measured on T1 enhanced magnetic resonance images of the brain from the axial, coronal, and sagittal aspects; the product of these values was divided by 2 to determine the tumors' volumes (19). Neurosurgeons and imaging experts evaluated the surgical records, intraoperative videos, and postoperative imaging data to determine the degrees of resection. The criteria for complete resection of CNs were 100.0% macroscopic resection of the tumor, the others were defined as incomplete resection (15).

### Statistical Analyses

IBM®SPSS® software, version 25.0 (IBM Corporation, Armonk, NY, USA) was used for the statistical analyses, and GraphPad Prism software, version 7 was used for mapping and statistical analyses (GraphPad Software, San Diego, CA, USA). Binary logistic regression analyses were performed. OS was determined from the date of surgery until the date of death or the last follow-up assessment. PFS was calculated from the date of surgery until the date of tumor recurrence or the last follow-up assessment. The progression dates were determined retrospectively based on the magnetic resonance imaging (MRI) findings or symptoms consistent with tumor progression. The Kaplan-Meier method was used to evaluate the OS and local tumor control rates. The risk factors associated with mortality after treatment were identified using Cox proportional hazards regression models and multivariate analysis.

## Results

### Patients' Characteristics

This was a single-center study that reviewed clinical data from 67 patients with CN who underwent surgery at Capital Medical University's Sanbo Brain Hospital. The study group had a mean ± standard deviation (SD) age of 30.60 ± 10.67 years and comprised 39 men and 28 women, with a male-to-female ratio of 1:0.72 (Table 1).

**Table 1:** Demographic, clinical, and treatment characteristics of 67 patients with central neurocytoma.

**Variable**	***N* (%)**
**Age, mean years (range)**	30.60 ± 10.67 (19–74)
<50 years	63 (94.0%)
≥50 years	4 (6.0%)
**Gender**	
Male	39 (58.2%)
Female	28 (41.8%)
**Preoperative KPS average ± SD**	78.46 ± 9.23
Functionally dependent group (KPS < 70)	26 (38.8%)
Functionally independent group (KPS ≥ 70)	41 (61.2%)
**Reason for treatment**	
Headache	47 (70.1%)
Nausea and vomiting	16 (23.9%)
Limb weakness	13 (19.4%)
Visual deficit	11 (16.4%)
Incidental finding	7 (10.4%)
Seizures	2 (3.0%)
Hearing lost	2 (3.0%)
Memory disturbance	2 (3.0%)
Disturbance of consciousness	1 (1.5%)
**Imaging characteristics**	
Gross tumor volume mean ± SD (range)	42459.96 ± 41475.99 (1579.47–196000.00) mm^3^
Cystic solid tumor	50 (74.6%)
Solid tumor	17 (25.4%)
Unilateral ventricle	33 (49.3%)
Both lateral ventricles	34 (50.7%)
Third ventricle involved	45 (67.2%)
**Pathological features**	
GFAP positive	16 (23.9%)
Olig-2 positive	7 (10.4%)
P53 positive	29 (43.3%)
PTEN positive	22 (22/33, 66.7%)
Median Ki-67 index (range)	5.0% (1.0%-20.0%)
**Treatment modalities**	
Complete resection	55 (82.1%)
Surgery alone	31 (46.3%)
Surgery and RT followed	24 (35.8%)
Incomplete resection	12(17.9%)
Surgery alone	3(4.5%)
Surgery and RT followed	9(13.4%)

The most common symptoms were headache (47/67; 70.1%) accompanied by nausea and vomiting (16/67; 23.9%), followed by limb movement disorders (13/67; 19.4%) and sensory disorders (3/67; 4.5%). Eleven patients (16.4%) had impaired vision, 2 (3.0%) experienced the onset of epilepsy, and 2 (3.0%) experienced memory impairment. Seven patients had no symptoms or signs during the physical examinations. The patients' mean KPS score was 78.46 ± 9.23 on admission.

### Pathologic Evaluations and Immunohistochemistry

Sixty-seven patients were diagnosed with World Health Organization (WHO) grade II CN by 3 pathologists. The median value of Ki-67 was 5.0%, ranged from 1.0 to 20.0%. There were 10 cases of necrosis, 7 cases of higher levels of mitotic activity (≥3 mitoses/10 high power fields), 2 cases of vascular proliferation and 1 case of nuclear atypia. Fifty-one patients were diagnosed with atypical CN. Sixteen cases (23.9%) were GFAP positive.

### Imaging Characteristics

**Figure 1:**
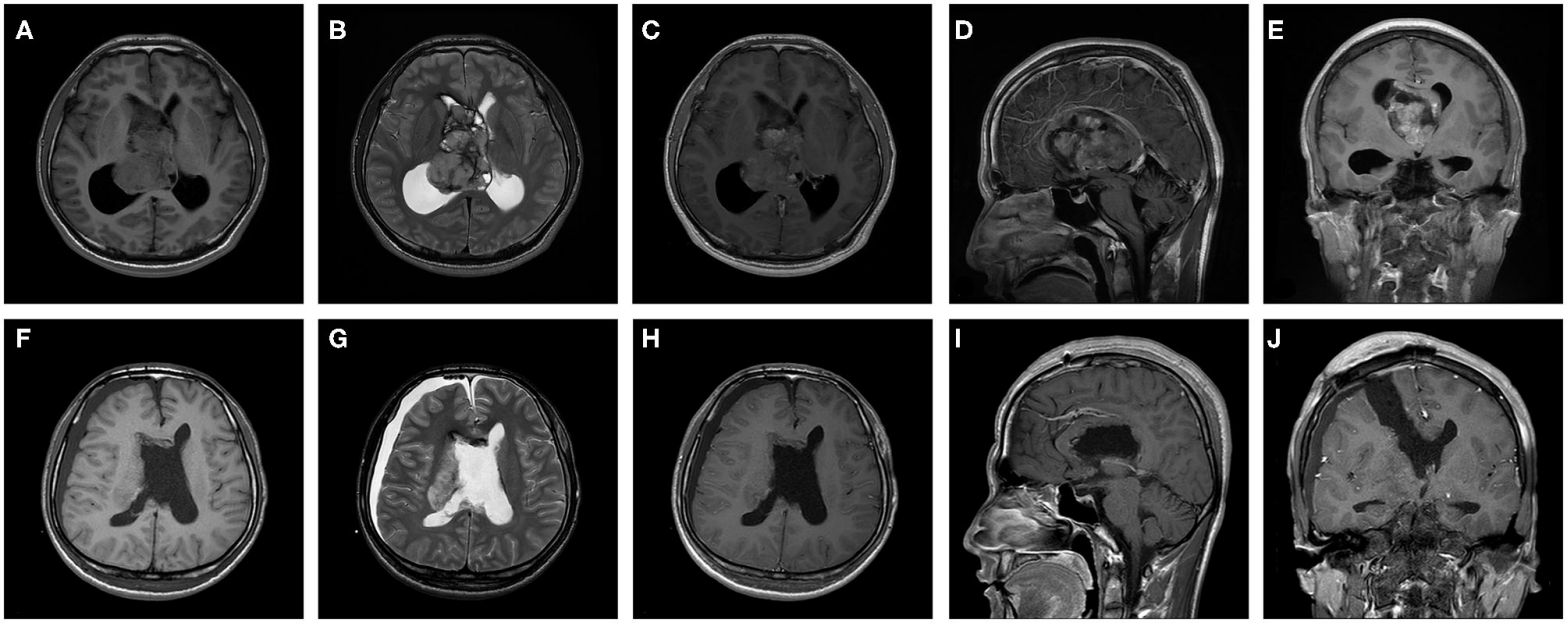
A 27-year-old male patient with central neurocytoma in treatment with surgery. Preoperative **(A)** T1 weighted axial sequence, **(B)** T2-weighted axial sequence, **(C)** T1- enhanced axial sequence, **(D)** T1-enhanced sagittal sequence, and **(E)** T1-enhanced coronal sequence MRI scans showing a 64.7 × 44.8 × 49.4 mm-sized mass in bilateral lateral ventricles and third ventricle and marked ventricular dilatation. Postoperative **(F)** T1 weighted axial sequence, **(G)** T2-weighted axial sequence, **(H)** T1-enhanced axial sequence, **(I)** T1-enhanced sagittal sequence and **(J)** T1-enhanced coronal sequence MRI scans indicating that CN was completely excised and ventricles return to normal slightly.

An evaluation of the patients' preoperative MRI and computed tomography data showed that the mean ± SD CN volume was 42,459.96 ± 41,475.99 mm3 (range, 1,579.47–196,000.00 mm3), 50 patients (74.6%) had cystic solid tumors, and 28 patients (41.8%) had calcification. All CNs were located in the lateral ventricles and 45 patients' (67.2%) tumors had invaded the third ventricle. After a comprehensive assessment, 55 patients with CN received the treatment of complete resection of tumor and the patient's hydrocephalus was relieved (Figure 1). The entire group does not appear in patients with tumor of craniospinal dissemination.

### Analysis of Treatment

All patients underwent surgery; the suprafrontal sulcus approach was used for 62 patients (62/67, 92.5%) and the transcallosal approach was used for 5 (7.5%). Fifty-five patients (82.1%) underwent complete and 12 patients (17.9%) underwent incomplete resections. Among the patients who underwent complete resections, 24 (24/55, 43.6%) received postoperative radiotherapy. Among the 12 patients (9/12, 75.0%) who underwent incomplete resections, 9 (9/12, 75.0%) received postoperative radiotherapy. For 3 of those patients, radiotherapy was not offered because of their poor health status (*n* = 2) and personal reasons (*n* = 1).

### Survival Analysis

In this study, 67 patients were followed for 0.5–135 months (mean, 43.5 ± 35.4 months). The mean ± SD 5 and 10 year OS rates were 84.8% ± 6.1%, and the mean ± SD 5 and 10 year PFS rates were 86.5% ± 7.6% and 79.8% ± 9.5%, respectively. Seven patients died, of whom 2 died as a consequence of tumor recurrences, and the other causes of death were epilepsy, cerebrovascular accidents, and unknown. The survival duration ranged from 0.50 to 135.00 months. Four patients with atypical CN had tumor recurrences *in situ*, and the mean time to tumor progression was 62.25 ± 17.05 months (range, 35–82 months); these patients included 2 who underwent complete resections and whose Ki-67 index were 10.0%, 2 who underwent incomplete resections and received adjuvant radiotherapy after surgery.

**Figure 2:**
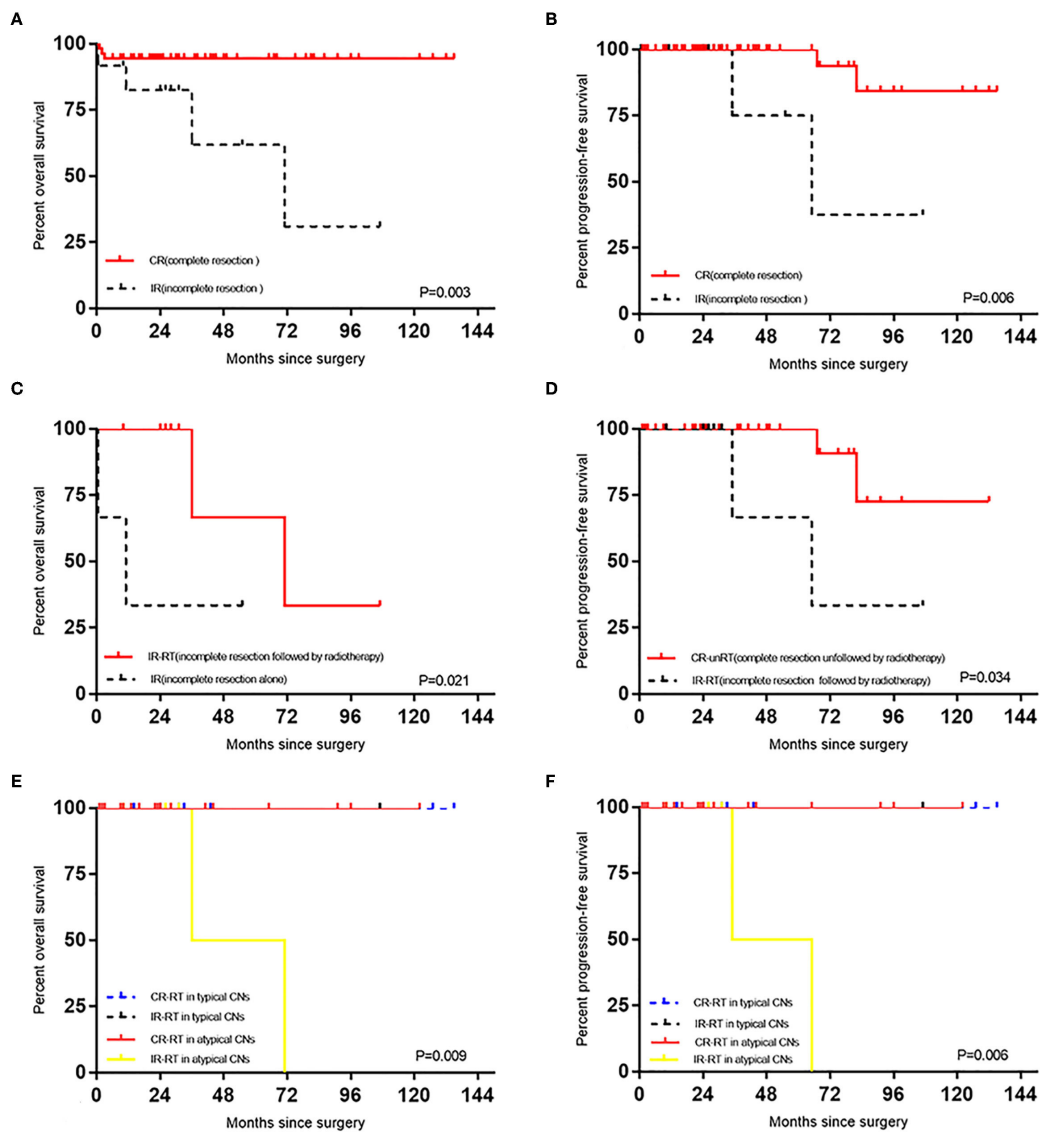
**(A)** Effect of degree of surgical resection on OS in patients with CN. **(B)** Effect of degree of surgical resection on PFS in patients with CN. **(C)** Effect of radiotherapy on OS in patients with CN after incomplete resection. **(D)** The difference of PFS between the CR-unRT and the IR-RT. **(E)** Survival stratified by current classification of CN, OS curves of CN patients with different treatments. **(F)** Survival stratified by current classification of CN, PFS curves of CN patients with different treatments.

The complete resection group's mean OS duration (127.61 ± 4.15 months) was significantly longer than the incomplete resection group's mean OS duration (63.54 ± 14.61 months) (*p* = 0.003) (Figure 2) and (**Table 3**). The complete resection group's mean PFS duration (125.78 ± 6.05 months) was significantly longer than the incomplete resection group's mean PFS duration (73.25 ±15.68 months) (*p* = 0.006) (Figure 2) and (**Table 3**). In the complete resection group (including atypical CN), postoperative radiotherapy had no effect on OS (*P* = 0.129) and PFS (*P* = 0.233), but in the incomplete resection group, postoperative adjuvant radiotherapy could significantly prolong the survival period of patients (*P* = 0.021, mean: 71.33 ± 16.74 months vs. 17.88 ± 10.88 months) (Figure 2), and there was no significant effect on PFS (*P* = 0.414). The mean PFS duration among patients in the complete resection group who did not receive radiotherapy was significantly longer than that among patients in the incomplete resection group who received radiotherapy (117.00 ± 9.42 months vs. 69.00 ± 17.05 months; *p* = 0.034) (Figure 2). The mean OS duration did not differ between the patients in the complete resection group who did not receive radiotherapy and those in the incomplete resection group who received radiotherapy (*p* = 0.263). Compared with the incomplete resection of atypical CNs, complete tumor resection significantly prolonged the mean ± SD OS (116.46 ± 3.83 months vs. 53.50 ± 17.50 months) (*p* = 0.013) and PFS (112.18 ± 6.24 months vs. 50.00 ± 15.00 months) durations (*p* = 0.00000007). Stratification analysis for survival according to the current classification criteria of CN, patients with atypical CN had relatively shorter OS and PFS (*p* = 0.009 and *p* = 0.006, respectively; Figure 2) after treatment of incomplete resection of tumor in the postoperative RT group. A similar analysis could not be performed due to insufficient the number of patients with typical CN in subgroups. The recurrence rate of atypical CN (7.8%) was higher than that of typical CN (0.0%), and adjuvant radiotherapy had no effect on OS (*p* = 0.241) or PFS (*p* = 0.302) after the complete resection of atypical CN. The mean ± SD PFS duration was significantly shorter for patients aged ≥ 50 years (54.60 ±15.68 months) than for those aged < 50 years (125.61 ± 5.09 months) (*p* = 0.00028) (**Table 3**). The multivariate analysis showed that incomplete tumor resection (odds ratio [OR], 10.736; 95% CI, 1.948–59.177; *p* = 0.0064) was associated with an increased risk of mortality, and that incomplete tumor resection substantially increased the risk of tumor recurrence (odds ratio [OR], 9.864; 95% CI, 1.358–71.625; *p* = 0.024).

### Functional Evaluations

Based on a retrospective evaluation of the medical records, the admitted patients were separated into 2 groups, namely, the functionally dependent group (KPS < 70) and the functionally independent group (KPS ≥ 70)(20); the patients' mean ± SD KPS score was 78.46 ± 9.23. Twenty-six patients (38.8%) were functionally dependent. Patients in the independent group presented less favorable survival outcomes than did patients in the dependent group (*p* = 0.003, mean: 123.82 ± 3.14 vs. 90.27 ± 15.59 months) (**Table 3**). A multivariate Cox model analysis showed that functional dependence was associated with an increased risk of mortality (OR, 12.466; 95% CI, 1.480–105.015; *p* = 0.020).

**Figure 3:**
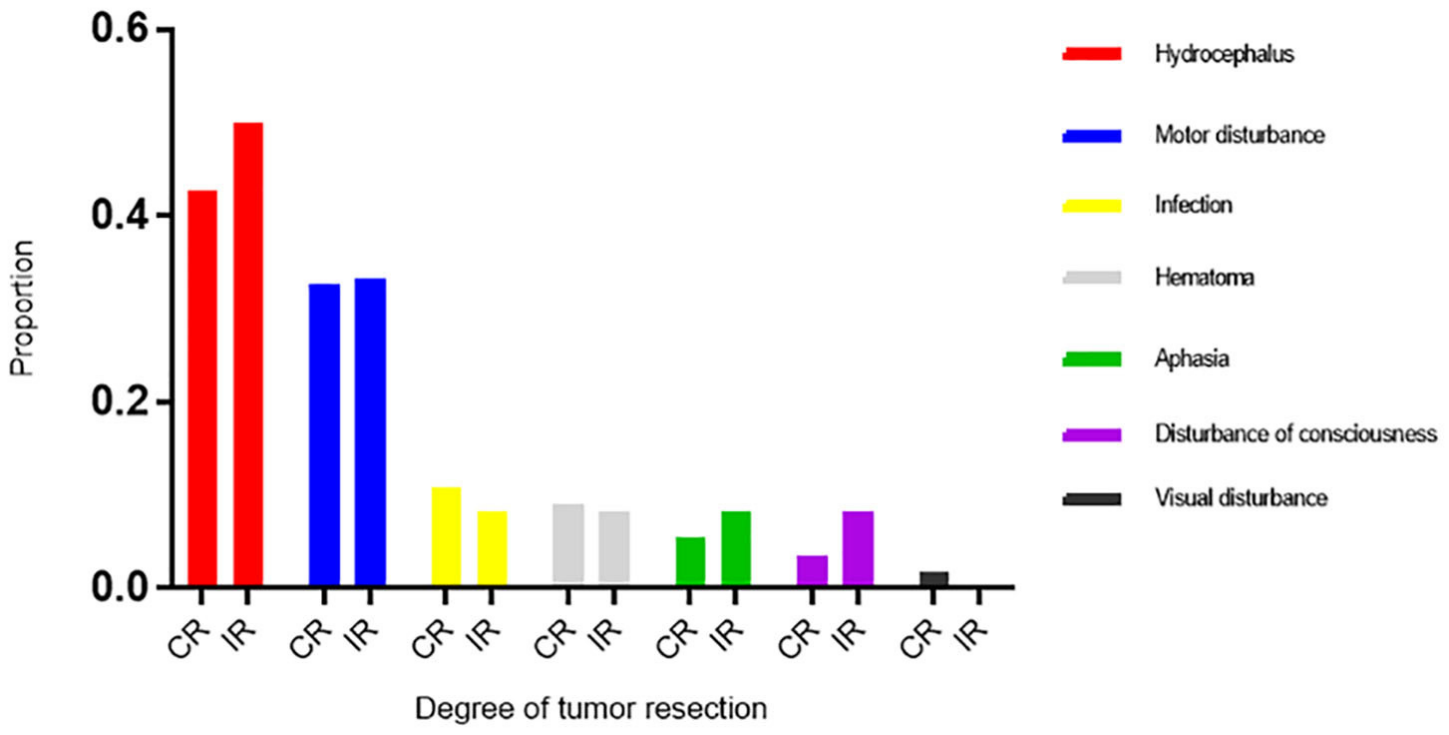
The proportion of perioperative complications in CR and IR groups.

The perioperative complications included hemorrhage, hydrocephalus, infection, dyskinesia, disturbances of consciousness, visual disturbances, aphasia, and epilepsy (Table 2). Twenty-two patients (32.8%) had dyskinesia, of whom 7 had dyskinesia before surgery. Twenty-nine patients (43.3%) had hydrocephalus after surgery, of whom 9 required external drainage and 4 required ventriculoperitoneal shunts. Three patients had disturbances of consciousness after surgery, and they survived 0.5–10 months. The amount of tumor resected and postoperative hemorrhage, hydrocephalus, infection, dyskinesia, disturbances of consciousness, aphasia, and epilepsy were not correlated (Table 2). Compared with the incomplete resection group, the complete resection group had a higher frequency of infection (10.9% vs. 8.3%), and hemorrhage (9.1% vs. 8.3%) rates, and a lower rate of hydrocephalus (41.8% vs. 50.0%) (Figure 3).

**Table 2:** Perioperative complications and functional outcomes at last follow-up.

**Variable**	***N* (%)**	***P*-value**		
		**Surgery approaches**	**Removal levels**	**Postoperative radiotherapy**
Hydrocephalus	29 (43.3%)	0.060	0.717	
Motor disturbance	22 (32.8%)	0.497	0.933	
Infection	7 (10.5%)	0.469	0.748	
Hematoma	6 (9.0%)	0.506	0.891	
Aphasia	4 (6.0%)	0.597	0.728	
Disturbance of consciousness	3 (4.5%)	0.647	0.507	
Visual disturbance	1 (1.5%)	0.796	0.632	
KPS at last follow-up				
100–90	43 (64.2%)			
90–80	7 (10.5%)			
80–70	8 (11.9%)			
<70	9 (13.4%)	0.206	0.920	0.281
Motor weakness at last follow-up	11 (16.4%)	0.062	0.090	0.783
Hydrocephalus at last follow-up	6 (9.0%)	0.603	0.026	0.414
Speech disturbance at last follow-up	3 (4.5%)	0.655	0.480	0.573
Memory deficit at last follow-up	3 (4.5%)	0.654	0.476	0.537
Seizure at last follow-up	2 (3.0%)	0.718	0.230	0.983

During follow-up, 17 patients (25.4%) were functionally dependent and 11 patients (16.4%) had dyskinesia (Table 3). Perioperative dyskinesia increased the risk of long-term dyskinesia by 28.385 times (*P* = 0.002). It should be noted that only 6 patients (9.0%) had hydrocephalus at the follow-up node, 3 patients (3/6, 50.0%) had incomplete resection. One patient died of herniation caused by hydrocephalus. Of the 5 patients who survived hydrocephalus, 3 required ventriculoperitoneal shunts at our hospital, and 1 required extraventricular drainage. The hydrocephalus risk in incomplete group increased by 8.833-fold (*p* = 0.026) during follow-up. There was no correlation between limb movement disorders and radiotherapy (*p* = 0.783). Memory impairment and postoperative radiotherapy were not correlated (*p* = 0.537). However, the incidence of memory impairment in the postoperative adjuvant radiotherapy group was higher than that in the surgery alone group (6.1% vs. 2.9%). Significantly higher proportions of patients in the complete resection group had limb dysfunction (20.0% vs. 0.0%) than did those in the incomplete resection group. The choice of operation mode had no effect on the memory of the patients with CN in the ventricle at the follow-up node (*p* = 0.654). Of the 4 patients treated using the transcallosal approach, 2 had perioperative memory impairments and 2 patients' memory impairments were restored to normal during follow-up. Three patients, of whom 2 had received postoperative adjuvant radiotherapy, had memory impairments; their operations involved the superior frontal sulcus approach. Memory impairment was considered to be related to radiotherapy and to damage to the fornix during surgery.

**Table 3:** Analysis of part of prognosis factors for 67 central neurocytoma (CN) in 5-years Progression-Free Survival (PFS) and Overall Survival (OS).

**Variable**	**OS (%)**	***P*-value**	**PFS (%)**	***P*-value**
**Age**		0.382		0.00028
<50 years	90.1 ± 4.4		97.0 ± 3.0	
≥50 years	75.0 ± 2.2		80.0 ± 17.9	
**Removal degrees**		0.003		0.006
Complete resection	94.4 ± 3.1		100.0 ± 0.0	
Incomplete resection	61.9 ± 19.8		75.0 ± 21.7	
**Post-operative RT^a^**		0.320		0.780
Yes	92.3 ± 7.4		92.3 ± 7.4	
No	85.0 ± 6.2		100.0 ± 0.0	
**Type**		0.258		0.204
Typical CN	81.3 ± 9.8		100.0 ± 0.0	
Atypical CN	92.2 ± 4.6		96.0 ± 3.9	
**Karnofsky performance status score (KPS)**		0.003		0.191
<70	71.7 ± 12.8		85.7 ± 13.2	
≥70	97.4 ± 2.5		92.3 ± 7.4	

a *RT, radiotherapy*.

## Discussion

CN is a rare primary intracranial tumor that is classified as a WHO grade II tumor. However, about 25.0% of these tumors are atypical CNs, which may be more aggressive (1, 14, 18, 21, 22). CN treatment includes surgical resection and radiotherapy (15). This study aimed to clarify the best treatment plan for patients with CN.

Many investigators believe that the prognosis of patients who have undergone complete resection is better than that of patients who have undergone incomplete resection (8, 15, 23, 24); the initial surgical treatment can provide long-term local control and reduce the risk of permanent functional defects (8). The results from this study showed that complete tumor resection could significantly prolong the OS and PFS durations for patients with CN, while incomplete tumor resection considerably increased the risks of death and tumor recurrence.

Treatment of benign or atypical CNs that comprises adjuvant radiotherapy after complete or incomplete tumor resection is widely accepted currently (14, 22, 25). In this study, 43.6% (24/55) and 75.0% (9/12) of the patients received adjuvant radiotherapy after complete and incomplete tumor resections, respectively. The results showed that radiotherapy did not affect the prognosis of the patients who had undergone complete tumor resections, and that radiotherapy could significantly prolong the OS duration for the patients who had undergone incomplete tumor resections. In the classification criteria adopted in this study, the cutoff value of KI-67 is relatively low, and it is undeniable that the index of KI-67 is affected by many factors (26). Similarly, adjuvant radiotherapy had no effect on OS among the patients who had undergone complete resections of atypical CNs. In the presence of postoperative radiotherapy, those patients with a atypical CN treated with incomplete resection had worse prognosis, while those patients with atypical CN treated with complete resection and those with a typical CN (regardless of the degree of resection) had comparable prognosis. Adjuvant radiotherapy can cause side-effects (12, 16, 27); therefore, it is not recommended after the total resection of CNs. At our center, radiotherapy is recommended for patients who have undergone incomplete tumor resections; this concurs with the recommendations from other centers (7, 22). Some authors have even suggested that patients who receive adjuvant radiotherapy after incomplete CNs resections could have the same prognoses as patients who have undergone complete tumor resections (28). However, this study shows that the PFS of patients in this group is lower than that of patients who undergo incomplete resection followed by radiotherapy. Therefore, complete resection may improve the OS and PFS of patients with CN to a greater degree. In addition, this study is the first to show that age ≥ 50 years is independently associated with reduced OS rates among patients with CN. Therefore, for elderly patients, clarification is required regarding the inclusion of other treatments, which could be achieved by undertaking studies involving larger sample sizes.

The main perioperative complications were hydrocephalus and dyskinesia. Although fewer patients had postoperative disturbances of consciousness, these could greatly increase the risk of death. The findings from a retrospective survey of 82 patients from 23 institutions showed that the incidence of complications in the total resection group was similar to that in the subtotal resection group (11). This study also showed that the degree of tumor resection was not related to postoperative complications, but the frequency of infection, and bleeding in the incomplete resection group was relatively high, which may be related to the injury of the tissue around the contralateral ventricle during the total resection of the tumor. Interestingly, complete resections may reduce the risk of hydrocephalus. During follow-up, the incidence of hydrocephalus was much lower than that in the perioperative period, which may be associated with tumor removal and unblocking the cerebrospinal fluid circulation pathway (27, 29). Moreover, during the early operations undertaken at our center, some patients may have experienced unilateral ventricular dilatation and hydrocephalus postoperatively. With the improvement of the surgery plan, such as septostomy of the septum pellucidum or even third ventriculostomy, the probability of hydrocephalus will be reduced. Third ventriculostomy could be relatively difficult in some patients with insignificant dilatation of the interventricular foramina, endoscopic assistance may help solve this problem. But it is not allowed to operate forcibly to achieve the purpose, so as to avoid damaging important structures such as forniceal columns, vena septi pellucidi, etc.

Functional dependence on admission is an independent risk factor associated with mortality and long-term survival in patients with CN. The preoperative KPS score was low, which was associated with the invasion of the basal ganglia. Although some patients recovered following exercise and rehabilitation, 16.4% of the patients continued to experience physical disabilities, which reduced their quality of life.

Applying radiation to particular parts of the brain, including the hippocampus, may damage long-term memory (30). In this study, 44.6% of the patients received postoperative adjuvant radiotherapy, but this was not directly related to limb movement disorders and memory impairment. Nevertheless, the proportions of patients with memory impairment was higher in the postoperative radiotherapy group; this does not rule out the possibility that the amount of tissue resected and the operative approach may have adversely affected the patients' memories (31–33). However, Kim et al. did not find any differences between surgical methods in relation to the KPS scores or surgical complications, including memory loss, motor weakness, and speech impairment (15). Generally, the choices of operation for CN is transcortical approach. Because CNs mostly expand and compress to the contralateral direction, so ostomy is designed to a relatively medial domain of the cerebral cortex. The length of the ostomy was prolonged according to the length of the tumor via superior frontal sulcus, and the incision was mostly on the prefrontal side due to the tumor usually blocking the interventricular foramen. The retractor was used throughout the operation to prevent subdural or epidural hematoma caused by brain tissue collapse. After the CN was exposed, it was separated along the boundary and protected by cotton pieces to prevent hemorrhage from entering the ventricle and to prevent tumor seeding and planting (there were little reports on this). The texture of the CN is usually soft, however, the blood supply is more abundant and from striata arteries, pars postcommunicalis, and choroidal artery in general, it is necessary to protect the thalamoperforating arteries during the operation, which supplies the basal ganglia and thalamus. This study also showed that the different surgical approaches do not affect the patients' memories during follow-up. The length of the corpus callosum incision is <2 cm during the operation, it is hoped that this approach will reduce cognitive and speech impairments in patients based on our institutional experience.

Some studies' findings have shown that the proliferative potential of CN may significantly affect patients' prognoses (34, 35). Although previous studies' findings have suggested that atypical relatively CNs are associated with shorter OS and PFS intervals (4, 18, 21, 22), the results from this study showed that there were no differences between the atypical CN group and the typical CN group regarding the OS and PFS durations, and no craniospinal dissemination occurred. In addition, complete tumor resection could prolong OS and control local recurrences in patients with atypical CN. However, the atypical CN recurrence rate was higher than that noted with typical CN (7.8% vs. 0.0%). During follow-up, 2 patients' tumors, which had Ki-67 indexes of 10.0%, recurred after complete tumor resections, which suggests atypical CNs require particular attention.

In summary, CNs should be resected completely and this should be the first choice of treatment for CNs, which will reduce the risk of postoperative hydrocephalus. Following incomplete resections, adjuvant radiotherapy can result in relatively good prognoses. Patients aged ≥ 50 years should be monitored closely. The significance and value of classifying atypical CNs histologically require further investigation. This was a single-center study involving a large sample, but multicenter, prospective studies with longer-term follow-up intervals are required to develop a standardized treatment plan.

## Conclusions

On the premise of protecting function as much as possible, the first choice of treatment for CN should be complete resection; adjuvant radiotherapy is not necessary after complete resection. For patients who have undergone incomplete tumor resections, postoperative adjuvant radiotherapy improves patients' prognoses. Adjuvant radiotherapy is not recommended following the complete resection of atypical CNs, but rigorous follow-up assessments that involve imaging are necessary. The findings of this study will help guide decision-making regarding the treatment of CNs and will maximize the benefits of treatment for patients with CN.

## Data Availability Statement

The clinical datasets generated during and/or analyzed during the current study are available from the corresponding authors on reasonable request.

## Ethics Statement

The studies involving human participants were reviewed and approved by the Ethics Committee of Sanbo Brain Hospital. The patients/participants provided their written informed consent to participate in this study.

## Author Contributions

CYa and CYu designed the study. SH, ZY, and YY collected the data and revised the manuscript for important intellectual content. XQ performed the histological examination of the samples. SH and ZY analyzed the data and wrote the article. All authors have read and approved the final manuscript.

## Conflict of Interest

The authors declare that the research was conducted in the absence of any commercial or financial relationships that could be construed as a potential conflict of interest.
